# Pathological Tumour Volume Percentage as a Quantitative Biomarker of Biological Aggressiveness in High-Risk Prostate Cancer

**DOI:** 10.3390/cancers18071069

**Published:** 2026-03-25

**Authors:** Lorand Tibor Reman, Călin Chibelean, Daniel Porav-Hodade, Árpád-Olivér Vida, Ciprian Todea Moga, Veronica Maria Ghirca, Raul-Dumitru Gherasim, Rares-Florin Vascul, Orsolya-Brigitta Katona, Szabolcs Andre, Edva Anna Frunda, Orsolya Katalin Ilona Martha

**Affiliations:** 1 Department of Urology, George Emil Palade University of Medicine, Pharmacy, Science, and Technology of Targu Mures, 540139 Targu Mures, Romania; tibor.reman@umfst.ro (L.T.R.); daniel.porav-hodade@umfst.ro (D.P.-H.); arpad.vida@umfst.ro (Á.-O.V.); ciprian.todea@umfst.ro (C.T.M.); maria.ghirca@umfst.ro (V.M.G.); raul-dumitru.gherasim@umfst.ro (R.-D.G.); orsolya.martha@umfst.ro (O.K.I.M.); 2 Department of Urology, Targu Mures, Clinical Hospital, Piata Bernady Gyorgy, Nr. 6, 540072 Targu Mures, Romania; andre.szabolcs.25@stud.umfst.ro; 3 Institution Organizing University Doctoral Studies (I.O.S.U.D.), George Emil Palade University of Medicine, Pharmacy, Science, and Technology of Targu Mures, 540139 Targu Mures, Romania; vascul.rares-florin.25@stud.umfst.ro (R.-F.V.); katona.orsolya-brigitta.25@stud.umfst.ro (O.-B.K.); frunda.anna@gmail.com (E.A.F.); 4 Department of Anaesthesiology and Intensive Care, County Emergency Clinic Hospital of Targu Mures, 540139 Targu Mures, Romania

**Keywords:** high-risk prostate cancer, tumour volume percentage, radical prostatectomy, risk stratification

## Abstract

**Background**: Tumour volume percentage (TVP) is considered an important pathological parameter, particularly in prostate cancer, representing the ratio of tumour volume to the total gland, and it can be used to measure the quantity of malignancy. Previous reports have already demonstrated that an elevated tumour volume percentage is associated with unfavourable factors, including extraprostatic extension, positive surgical margins, and lymph node metastasis. The independent value of TVP, especially in high-risk prostate cancer treated by radical prostatectomy, remains an area of active research, despite established prognostic factors such as PSA, ISUP grade, and TNM stage. **Materials and Methods**: We retrospectively analyzed the records of 159 high-risk prostate cancer patients who underwent open or laparoscopic radical prostatectomy between January 2016 and January 2025 at the Clinic of Urology of Targu Mures. High-risk patients were defined as those with ISUP grade 4–5 or PSA >20 ng/mL or clinical stage ≥T2c or stage cT3–4 and/or lymph node metastasis. Tumour volume percentage was calculated from the final pathology result and was determined as the proportion of prostate cancer volume relative to the total prostate volume. Clinical and pathological features, including PSA, ISUP grade, TNM stage, surgical margin, and lymph node involvement, were reported. To assess TVP as an indicator of tumour aggressiveness, univariate and multivariate regression analyses were performed. A *p*-value < 0.05 was considered statistically significant. **Results**: A total of 159 high-risk prostate cancer patients (100%), with a median age of 66 years, who underwent open or laparoscopic radical prostatectomy were included. The median tumour volume percentage was 7.6%, and the median prostate volume was 43.8 cc. On univariate analysis, patients with extraprostatic extension (*p* < 0.001), positive surgical margins (*p* = 0.005), a higher ISUP grade (*p* < 0.001), and lymph node metastasis (*p* = 0.006) exhibited higher TVP compared to their counterparts. A significant correlation was also observed between TVP and the number of positive biopsy cores (*p* < 0.001), a higher PSA value (*p* = 0.005), and a younger age (*p* = 0.041). Conversely, no correlation was identified between TVP and perioperative factors such as hospital stay, surgery duration, ICU days, type of approach, or positive urine culture. Two regression models on multivariate analyses were performed with TVP as the dependent variable. In the continuous variable model (Adjusted R^2^ = 0.43, *p* < 0.001), independent predictors of higher TVP were the number of positive biopsy cores (B = 0.54, *p* < 0.001), the number of positive lymph nodes (B = 2.59, *p* < 0.001), and surgical margin dimension (B = 1.19, *p* < 0.001). Age, PSA, and perioperative variables showed no significant correlation with TVP on multivariate analysis. In the categorical regression model (Adjusted R^2^ = 0.438), statistical significance was confirmed (F-test, *p* < 0.001). Independent predictors of increased tumour volume percentage included ISUP grade 5 in the effect-coded model (B = +6.60, 95% CI: 0.96–12.25, *p* = 0.022), and pathological TNM stage pT4 (B = +24.70, 95% CI: 17.69–31.70, *p* < 0.001). ROC analysis showed limited-to-moderate discrimination for positive surgical margins (AUC = 0.655; 95% CI 0.565–0.744; *p* = 0.001) and stronger discrimination for pN1 (AUC = 0.793; 95% CI 0.650–0.936; *p* = 0.002). The Youden-derived cut-offs were 4.90% for positive surgical margins and 5.77% for lymph-node metastasis. **Conclusions**: Tumour volume percentage is significantly associated with several adverse pathological features in high-risk prostate cancer. Rather than a standalone biomarker, its association with adverse pathological features underscores its potential role in risk stratification models, and the incorporation into pathology reports and prognostic nomograms may improve clinical decision-making.

## Simple Summary

Current risk assessment of prostate cancer patients has many approaches, including the D`Amico classification, a multitude of nomograms, and the UCSF-CAPRA score, the majority of which rely on prostate-specific antigen levels, tumour grade, and tumour stage. In the case of high-risk prostate cancer patients, risk assessment often uses tumour volume percentage (TVP) as a correlate with adverse pathological features. TVP represents the proportion of tumour volume inside the prostate gland, and it can be used as an objective measurement of tumour burden and aggressiveness. In this study, we evaluated tumour volume percentage in the case of 159 high-risk prostate cancer patients treated by radical prostatectomy. We aimed to analyze whether a higher tumour volume percentage is associated with unfavorable pathological features such as positive surgical margins and lymph node metastasis. For the research community, these results highlight the potential of including TVP in standard pathology reports to contribute to the development of future risk stratification models.

## 1. Introduction

Prostate cancer remains the second most frequently diagnosed malignancy and the fifth cause of cancer death among men worldwide, with more than 1.460.000 estimated cases and 396.000 deaths in 2022 [1,2]. Risk stratification after radical prostatectomy is based on serum PSA [3], pathologic grade group [4], extraprostatic extension [5], seminal vesicle invasion [6], or surgical margin extent [7,8]. Prostate cancer is characterized by marked biological heterogeneity, ranging from indolent tumours that may never require treatment to aggressive diseases associated with early metastasis and cancer-specific mortality. According to the European Association of Urology (EAU) Guidelines, high-risk prostate cancer is defined as ISUP grade 4 or 5, or a PSA > 20 ng/mL, or a clinical stage greater than T2c determined by digital rectal examination, or evidence of lymph node involvement on cross-sectional imaging [9].

Treatment options for high-risk prostate cancer include watchful waiting for asymptomatic patients with a life expectancy <10 years, radical prostatectomy (RP) as part of potential multi-modal therapy associated with extended pelvic lymph-node dissection, and radiotherapy [10,11]. In a well-selected patient group diagnosed with locally advanced prostate cancer, radical prostatectomy with adjuvant or salvage treatment can yield long-term cancer control and a good survival rate [12]. Even though several treatment options are available for patients diagnosed with high-risk prostate cancer, a high recurrence rate is described regardless of the initial treatment approach [13]. Up to 50% of patients treated with radical prostatectomy experience biochemical recurrence (BCR), highlighting the limitations of current risk stratification tools, which rely on categorical variables such as pathological grade group, extraprostatic extension, or surgical margin extent [14].

Not all patients with high-risk prostate cancer experience a poor prognosis [15]. Previous studies have reported that patients with a Gleason score > 8 may have a recurrence-free survival rate of approximately 39% [16]. In this context, particularly among those with higher Gleason scores and consecutive high-risk disease, tumour volume becomes a critical determinant of outcome when the underlying tumour biology is already adverse [17]. TVP complements the Gleason score by providing a quantitative measurement of the total cancer burden and has been shown to predict advanced pathological stage, increased risk of biochemical recurrence, and an overall worse prognosis [18]. By contrast, the utility of PSA is limited, as it reflects not only the malignant tumour burden but may also be elevated in benign prostate hyperplasia [19] and acute or chronic prostatic inflammation [20].

The concept of tumour volume as a prognostic and staging measurement is already widely adopted in other solid organ malignancies, including kidney, testis, and breast cancer [21,22,23]. Tumour volume is an intuitive and easily measurable metric derived from final pathology specimens and, increasingly, from pre-operative imaging, such as mpMRI [24]. Recent work over the last decade has analysed TVP as an independent marker of adverse pathology, with nuanced results [25]. The analysis of tumour volume percentage as a prognostic factor, especially in high-risk prostate cancer treated by radical prostatectomy, remains an area of active research, despite established prognostic factors such as PSA, ISUP grade, and TNM stage [26]. Multiple contemporary series demonstrate that higher TVP was associated with adverse features, including extraprostatic extension, positive surgical margins, higher ISUP grade, and remains an independent risk factor related to biochemical recurrence rate in multivariate models. This has been shown whether tumour burden is expressed as absolute volume or as a ratio to the entire prostate gland [27].

The balance of recent evidence supports incorporating TVP into multivariable models and pathology reports, particularly to refine counselling on adjuvant therapy and surveillance intensity [19]. Ongoing standardization of measurements and harmonization of thresholds will be essential for broader adoption to achieve more precise, patient-specific risk stratification calculators. For future trials, it is important to identify patients at high risk for relapse who may benefit from aggressive adjuvant therapy, while excluding those in whom cancer could be amenable to curative monotherapy.

Tumour volume percentage represents a quantitative descriptor of intraprostatic tumour burden that integrates biological aggressiveness and spatial extent, yet its independent relevance in high-risk disease remains incompletely defined. In this context, we aimed to evaluate TVP as a pathological biomarker in a strictly defined high-risk prostate cancer cohort treated by radical prostatectomy with regional or extended pelvic lymph-node dissection. Specifically, we assessed the association of TVP with adverse pathological features and markers of metastatic potential. Our objective was to clarify whether TVP reflects intrinsic tumour biology beyond conventional categorical risk factors and whether it may contribute to refined postoperative risk stratification in high-risk prostate cancer.

## 2. Materials and Methods

We performed a single-center, retrospective study of 159 men (100%) who underwent open- or laparoscopic prostatectomy for clinically non-metastatic high-risk prostate cancer at the Clinic of Urology, Mures Clinic County Hospital. The study period spanned from January 2016 to January 2025. Data were extracted from the institutional database and the electronic medical records.

The inclusion criteria were the following: patients with biopsy-proven adenocarcinoma of the prostate, PSA ≥ 20 ng/mL or ISUP grade group 4/5 or cT2c (EAU high-risk prostate cancer criteria), treated by open- or laparoscopic prostatectomy. Patients with evidence of nodal or distant metastasis preoperatively, who had undergone salvage prostatectomy following prior neoadjuvant local- or systematic treatment, or if their records contained incomplete data (PSA, biopsy grade, pTNM or tumour volume metrics) were excluded.

All whole-mount prostatectomy specimens were handled according to standard protocols: inked, fixed in 10% neutral buffered formalin, and step-sectioned at well-established 3–4 mm intervals from the apex to the base, to produce whole-mount sections. Tumour foci were identified and mapped on the corresponding sections by a single, dedicated genitourinary pathologist to ensure consistency and eliminate inter-observer variability. Absolute tumour volume (TV, cc) was measured on the final specimen using a three-dimensional planimetric method. Tumour areas were outlined on each histological section and measured using planimetric techniques, and the cumulative tumour volume was obtained by summing the measured tumour areas across all sections and multiplying by the section thickness. Tumour volume percentage (TVP) was calculated as the ratio of absolute tumour volume to the total prostate gland volume, expressed as a percentage. All measurements were performed according to standardized institutional protocols to ensure consistency of pathological evaluation. Extraprostatic extension was defined as the presence of tumour cells in the periprostatic adipose tissue or beyond the prostatic capsule.

Patients with radiologically confirmed nodal or distant metastasis prior to surgery were excluded, while pathological lymph node involvement (pN1) identified after prostatectomy and lymph node dissection was recorded as a study outcome. Pelvic lymph node dissection (PLND) was performed at the time of radical prostatectomy. Based on the literature, pelvic lymph node dissection templates were categorized into limited and extended pelvic lymph node dissection [28], and it was executed according to the surgeon’s discretion. A limited PLND was defined as dissection primarily involving the obturator fossa with the external iliac nodes, while an extended PLND was defined as inclusion of the obturator, external iliac, internal iliac, and common iliac nodes [29]. Although pelvic lymph node dissection was categorized as limited or extended, the number of patients undergoing extended PLND was relatively small. Consequently, the number of pathologically confirmed nodal metastases within the extended subgroup was insufficient to allow for robust subgroup-level statistical analyses. Therefore, for statistical purposes, lymph node data from limited and extended PLND were analyzed together as a binary biological outcome. TVP was treated as a biological dependent variable, rather than an outcome surrogate.

Continuous variables were assessed for normal distribution via Shapiro–Wilk tests and visual inspection of histograms and Q–Q plots. Normally distributed variables are presented as mean ± SD, and non-normally distributed variables as median (IQR); categorical variables are presented as counts and percentages.

TVP was analyzed as a continuous variable. Associations between TVP and clinicopathological variables were first explored using univariate analyses. Continuous variables were assessed using Spearman’s rank correlation coefficient (ρ), while categorical variables were compared using the Mann–Whitney U test or Kruskal–Wallis test, as appropriate.

To identify independent predictors of TVP, multivariable linear regression models were constructed with TVP treated as a continuous dependent variable, including elements that demonstrated statistical significance in univariate analysis. Regression results were reported as β coefficients with corresponding 95% confidence intervals (CI). Model performance was evaluated using the F-test and the adjusted coefficient of determination (adjusted R^2^). Categorical predictors in multivariable models were parameterized using effect (deviation) coding; therefore, coefficients represent deviation from the overall mean rather than contrast to a single reference category.

Receiver operating characteristic (ROC) curve analysis was subsequently performed to evaluate the ability of TVP to correlate with adverse pathological outcomes, including positive surgical margins and lymph node involvement. The area under the curve (AUC) with 95% confidence intervals was calculated. For ROC threshold derivation, the Youden index was used. We did not perform internal validation (e.g., split-sample, cross-validation, or bootstrapping); hence, the reported cut-offs are exploratory and not intended for clinical decision-making.

A two-sided *p* < 0.05 was considered significant, and the analyses were performed in IBM SPSS Statistics (v26, Armonk, NY, USA).

Regression assumptions were evaluated before interpreting the model results. Residual distribution and homoscedasticity were assessed graphically, and multicollinearity among predictors was examined using variance inflation factors (VIF). All included variables demonstrated acceptable collinearity levels, and no violations of model assumptions were detected.

This study protocol was approved by the institutional ethics committee, which granted a waiver of informed consent for this retrospective analysis.

This study was conducted according to the guidelines of the Declaration of Helsinki and was approved by the local institutional ethics committee of Spitalul Clinic Judetean Mures (12852/09.10.2025). Informed consent was waived due to the retrospective nature of this study.

## 3. Results

A total of 159 patients met the inclusion criteria for high-risk prostate cancer and underwent radical prostatectomy between 2016 and 2025. Median age at surgery was 66 years (IQR: 50–76); median PSA at diagnosis was 13.2 ng/mL (IQR: 0.81–41). In 82 (51.5%) cases, the approach was open prostatectomy, while in 77 (48.5%) cases, a laparoscopic procedure was performed. Median time from the biopsy until the surgery was 78 days (IQR: 29–270). Operative metrics included a median operating time of 220 min (IQR: 150–360), an intensive care unit stay of 2 days (IQR: 1–6), and a median hospitalization of 6 days (IQR: 4–21). Pelvic lymph node dissection was performed during every surgery. In 11 cases (6.91%), nodal involvement was diagnosed. Positive surgical margins were described in 61 (38.3%) cases.

On biopsy histology, ISUP grade group distribution was as follows: ISUP 1 in 23/159 cases (14.4%), ISUP 2 in 97/159 (61%), ISUP 3 in 21/159 (13.2%), ISUP 4 in 12/159 (7.5%), and ISUP 5 in 6 of the patients (3.7%). All patients underwent ultrasound-guided transrectal prostate biopsy, with a median of 12 total cores obtained per patient (IQR: 6–21) and a median of 6 positive cores (IQR: 1–15). Perineural invasion was present in 48 (30.1%) and extraprostatic extension in 7 (4.4%) cases. Unilateral right-lobe prostate cancer was diagnosed in 27 (16.9%) cases, unilateral left-lobe involvement in 26 (16.3%) patients, and bilateral disease in 103 (66.6%) patients.

All 159 (100%) patients were classified as high-risk according to the EAU criteria. Although the median PSA was 13.2 ng/mL and the majority of biopsy specimens showed ISUP grade 1–2, these patients met the high-risk definition primarily through clinical staging. Specifically, 33 patients (20.75%) qualified based on PSA ≥ 20 ng/mL, 20 patients (12.5%) based on biopsy ISUP 4–5, and 128 patients (80.5%) based on clinical stage ≥ T2c. Clinical staging was determined through digital rectal examination (DRE) and confirmed by multi-parametric MRI (mpMRI) in accordance with institutional protocols. Because patients could meet more than one criterion, these categories are not mutually exclusive.

The pathological stage distribution was as follows: pT2c in 60/159 patients (37.7%), pT3a in 61/159 (38.3%), pT3b in 36/159 (22.6%), and pT4 in 2/159 (1.2%) cases. Overall, organ-confined disease (pT2c and negative margins) was observed in 33 (20.7%) patients, whereas non-organ-confined disease (≥pT3a) was present in 126 (79.3%) patients. On analysis of the prostatectomy specimen, ISUP grade 1 was identified in 6/159 (3.77%) cases, ISUP grade 2 in 94/159 (59.1%), ISUP grade 3 in 38/159 (23.8%), ISUP grade 4 in 4/159 (2.5%), and ISUP grade 5 in 17/159 (10.6%) patients. Compared with biopsy histology, ISUP downgrading on the prostatectomy specimen was observed in 39 cases, whereas upgrading occurred in 13 cases.

Based on the median nodal excision of 9 nodes (IQR: 6–32), lymphadenectomy in our cohort was predominantly consistent with a limited PLND (120/159), with a smaller proportion of patients undergoing extended dissection (39/159). In patients with limited PLND, a median of 7 (IQR: 5–13) nodes was dissected, with a node metastasis rate of 5% (4/120), while in the group of patients with extended PLND, the median number of excised nodes was 15 (IQR: 11–21), with a positivity rate of 18% (7/39). Combined pathologically confirmed nodal metastases (pN1) were identified in 11 patients (6.91%), with a median of 3 (IQR: 1–8) nodes. Because extended pelvic lymph node dissection yields a greater number of nodes and increases the likelihood of detecting nodal metastasis, the observed associations between tumour volume percentage and pN1 disease should be interpreted cautiously as they may partially reflect differences in nodal staging intensity.

Regarding the tumour involvement of the prostate on final exam, our findings were the following: bilateral in 147 (92.4%) cases and unilateral in 12 patients (7.6%), evenly distributed between left- and right-sided disease. The median prostate volume was 43.8 cc (IQR: 15–164), with a median tumour volume percentage of 7.6% (IQR: 0.1–32%). Patients’ demographics, clinical and tumour characteristics are summarized in Table 1.

Univariate analysis demonstrated that TVP was significantly associated with several established markers of prostate cancer aggressiveness. Associations between TVP and clinicopathological variables were evaluated using Spearman’s rank correlation for continuous variables and Mann–Whitney U or Kruskal–Wallis tests for categorical variables. Using Spearman’s rank correlation, a significant, albeit weak, negative correlation was identified between TVP and patient age (ρ = −0.162; 95% CI −0.314 to −0.002; *p* = 0.041). Conversely, TVP demonstrated significant positive correlation with higher preoperative PSA (ρ = 0.213; 95%CI, 0.060 to 0.365; *p* = 0.005) and the number of positive biopsy cores (ρ = 0.366; 95%CI, 0.227 to 0.504; *p* < 0.001), higher ISUP grade on both the biopsy and the final prostatectomy specimen (*p* < 0.001 and *p* < 0.001), presence of perineural invasion on biopsy (*p* < 0.001), and a greater number of positive lymph-nodes (*p* = 0.006), higher pT grade (*p* < 0.001) and presence of positive surgical margins (*p* = 0.005). The surgical approach (open vs. laparoscopic), total number of biopsy cores, and perioperative metrics (surgery duration, hospitalization days, and days in ICU) did not demonstrate a significant correlation with TVP.

**Figure 1:**
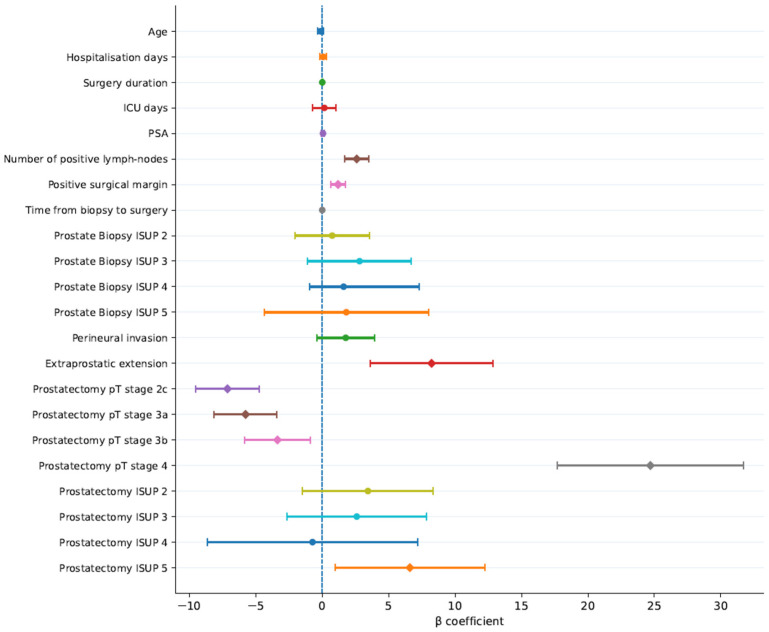
Forest plot of regression coefficients for factors associated with intraprostatic tumour burden in high-risk prostate cancer. Dots represent β coefficients and horizontal bars indicate 95% confidence intervals. The dashed vertical line indicates the null value (β = 0), with estimates to the right representing positive associations and those to the left negative associations. Pathological variables dominate over clinical and perioperative factors.

A multivariable linear regression model was performed to identify clinicopathological variables independently associated with TVP. The overall model was statistically significant (F-test *p* < 0.001) and explained approximately 44% of the variance in TVP (adjusted R^2^ = 0.438). Within the multivariable model, higher preoperative PSA levels, a greater number of positive biopsy cores, a higher pathological ISUP grade, and greater nodal burden were independently associated with increased TVP. Regression coefficients are presented as β coefficient estimates with corresponding 95% confidence intervals in Table 2. Among the evaluated variables, pathological stage pT4 demonstrated the strongest association with TVP (β = +24.70; 95% CI 17.69–31.70; *p* < 0.001). The presence of extraprostatic extension on biopsy was also significantly associated with higher TVP (β = +8.23; 95% CI 3.62–12.85; *p* = 0.001). Additionally, ISUP grade 5 on the final prostatectomy specimen was independently correlated with a significantly higher TVP (β= +6.60; 95% CI 0.96–12.25; *p* = 0.022). In contrast, perineural invasion and the surgical approach were not identified as independent factors of TVP in the multivariable model (Figure 1).

A second multivariable linear regression model was applied using continuous clinical and quantitative pathological variables, which also showed a significant overall fit (ANOVA *p* < 0.001). This model highlighted that three quantitative factors have continuously increasing, independent associations with TVP: number of positive lymph nodes (β = +2.59 per node; 95% CI 1.69–3.51; *p* < 0.001), number of positive biopsy cores (β = +0.54 per core; 95% CI 0.27–0.80; *p* < 0.001), and positive margin size (β = +1.19 per mm; 95% CI 0.63–1.76; *p* < 0.001). Clinical factors such as age, PSA, and all perioperative metrics were not independently associated with TVP after adjusting for these key pathological variables.

**Figure 2:**
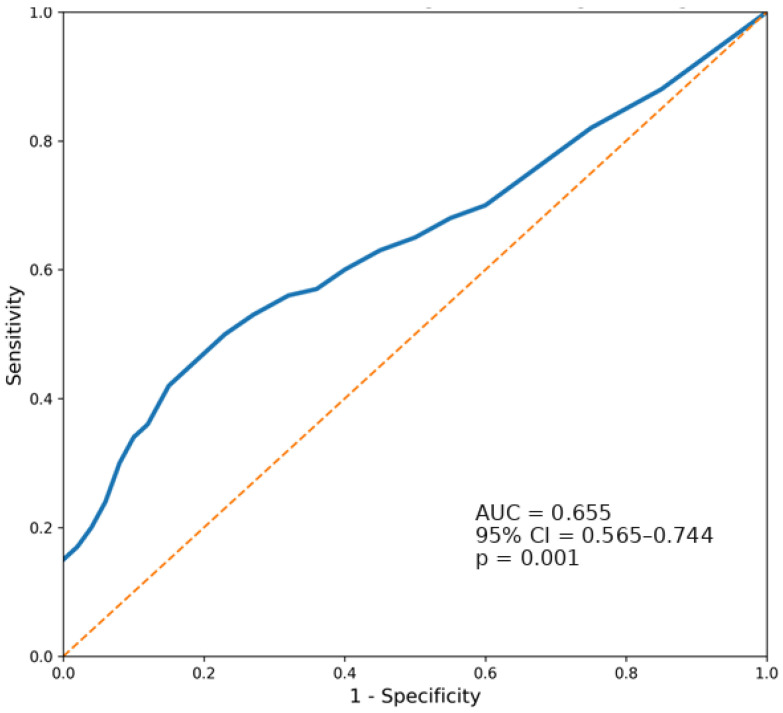
ROC curve evaluating the performance of tumour volume percentage (TVP) in predicting positive surgical margins. The blue line indicates the observed ROC curve, while the orange dashed diagonal line indicates the line of no discrimination.

**Figure 3:**
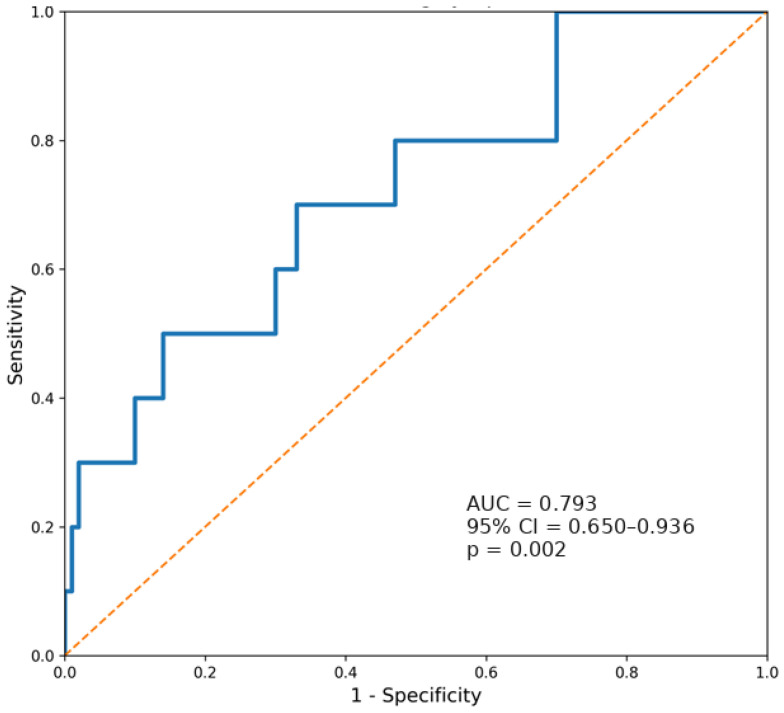
ROC curve evaluating the performance of tumour volume percentage (TVP) in predicting lymph node involvement. The blue line indicates the observed ROC curve, while the orange dashed diagonal line indicates the line of no discrimination.

Receiver operating characteristic (ROC) curve analysis was performed to evaluate the ability of TVP to discriminate adverse pathological outcomes following radical prostatectomy. TVP demonstrated a moderate discriminatory ability for identifying positive surgical margins, with an area under the curve (AUC) of 0.655 (95% CI 0.565–0.744; *p* = 0.001) (Figure 2). Using the Youden index, the optimal TVP threshold for identifying positive surgical margins was 4.90%, corresponding to a sensitivity of 67.2% and a specificity of 50.0%. A stronger discriminatory performance was observed for lymph node involvement, where ROC analysis yielded an AUC of 0.793 (95% CI 0.650–0.936; *p* = 0.002) (Figure 3). The optimal cut-off value for identifying nodal metastasis was 5.77%, providing a sensitivity of 80.0% and a specificity of 53.7%. These findings suggest that an increasing tumour volume percentage is associated with a higher likelihood of adverse pathological features, particularly nodal metastasis.

Overall, ROC analysis suggests that TVP has limited-to-moderate independent predictive value for local adverse pathology, including positive surgical margins, while demonstrating stronger performance in detecting nodal metastases. These results also support the incorporation of TVP into multivariate risk models rather than as a single prognostic marker. These cut-points should be interpreted as exploratory, hypothesis-generating thresholds derived from this single dataset; they were not internally validated and should not be used as a fixed clinical decision threshold.

## 4. Discussion

The objective of this study was not to evaluate long-term oncologic outcomes such as biochemical recurrence, metastasis-free survival, or overall survival, but rather to define TVP as a biological and pathological biomarker reflecting intrinsic aggressiveness at the time of surgery. By deliberately focusing on cross-sectional pathological endpoints, such as nodal metastasis, extraprostatic extension, and surgical margin extent, we avoid the confounding effects of adjuvant therapy, salvage treatment options, and heterogeneous follow-up protocols that disproportionately influence biochemical recurrence [30,31].

In this single-center 159 high-risk radical prostatectomy patients, we found that tumour volume percentage (TVP) aligns most strongly with markers of biological aggressiveness rather than perioperative or patient-related factors. On multivariate model analysis, higher TVP was independently associated with ISUP grade 5 on the prostatectomy specimen, extraprostatic extension, advanced local stage (pT4), a greater number of positive biopsy cores, larger positive surgical margins, and a higher number of lymph-node metastases; by contrast, age, PSA, duration of surgery, days in ICU, hospitalization days, and time from biopsy-to-surgery were not independently related to intraprostatic tumour volume percentage. Together, these findings indicate that TVP represents a quantitative marker of intraprostatic tumour burden associated with adverse pathological features at the time of surgery.

Our results align with recent multi-institutional and population-based evidence that tumour burden—expressed as absolute pathological tumour volume or as a percentage of the gland—co-varies with grade, stage, surgical margin extent, and is proportionally associated with worse prognosis [26]. In a recent analysis focused on high-risk disease, Raison et al. reported that pathological tumour volume is indeed an independent predictor of oncologic outcomes; however, its incremental prognostic value beyond standard clinicopathological factors was limited, underscoring a “useful but not decisive” role in risk models. The authors also noted that the feasibility of accurate preoperative TV assessment by MRI could still make TV a valuable preoperative risk marker, even though its post-hoc incremental value is modest [26]. This nuanced position resonates with our data: TVP tracks strongly with adverse pathology and remains significant in adjusted models, but the best discrimination emerges when TVP is combined with other strong predictors.

In a 2.394 patient scientific cohort, Yuk et al. demonstrated that both tumour volume and tumour-to-prostate volume ratio were associated with higher risk groups and independently predicted adverse outcomes on multivariate analyses, supporting tumour burden as an important risk factor [27]. Similarly, Baba et al. analyzed 557 men undergoing radical prostatectomy and identified an ROC-derived 2.8 cc. tumour-volume threshold that independently stratified biochemical recurrence, also noting the worse prognostic impact of posterior and peripheral-zone tumour location [25]. These findings are conceptually consistent with our observation that higher TVP was associated with adverse pathology and that cut-points can operationalize this risk factor in clinical workflows.

Not all studies, however, have found a uniformly independent or incremental role of TVP as a prognostic risk factor for prostate cancer patients. A study published in European Urology also classifies TV among core pathologic determinants. Early multivariable work highlighted associations between TV, Gleason score, stage, and margin extent in driving progression, anticipating risk model architectures in which TV-derived metrics complement grade and stage [32]. Our study mirrors these results: ISUP grade 5, pT4 stage, and margin extent were correlated with higher TVP, and the combined model that includes these risk factors yields the highest probability.

Related constructs, such as tumour-to-prostate ratio (tumour density), have also been explored. A work by Cho et al. suggests that scaling tumour burden to gland size may refine recurrence prediction in some settings, which conceptually aligns with our observation that TVP (the proportion) behaves as a compact marker of biological burden rather than a mere correlate of preoperative metrics [33]. These results are similar to our findings, and the consistent signal across these measures supports the centrality of volumetric burden in prostate cancer biology.

Clinically, these data affirm a pragmatic stance. TVP behaves as a biological risk marker: higher values correlate with high ISUP grade, extraprostatic extension, thicker positive surgical margins, and nodal spread [34]. For postoperative decision-making, TVP can strengthen discussions about adjuvant therapy when aligned with grade, stage, surgical margins, and positive lymph nodes [35]. Given the cross-sectional design of the present study and the absence of oncologic follow-up outcomes, TVP should currently be interpreted as a candidate variable for inclusion in postoperative risk models that require prospective validation before influencing therapeutic decision-making. For preoperative risk-stratification, growing radiologic accuracy in tumour volume estimation based on mpMRI could make preoperative tumour volume estimation a practical tool. Future work should test whether integrating preoperative MRI-derived TV into nomograms improves decision-making and whether standardized reporting of TVP adds consistency across centers. In this context, TVP may represent a quantitative descriptor of tumour burden that complements conventional categorical pathological variables. Upcoming studies integrating tumour volume percentage into multivariable prognostic models alongside grade, stage, surgical margin status, and nodal involvement may help clarify its incremental value for guiding postoperative management strategies.

Our findings suggest potential relevance for next-generation imaging interpretation based on artificial intelligence (AI) models. Accurate preoperative risk stratification increasingly depends on mpMRI, yet AI algorithms require robust pathological datasets to learn precise volumetric segmentation. By demonstrating that pathologically measured TVP can be correlated with nodal metastasis compared to standard clinical variables, our findings further support volumetric burden as a relevant feature for imaging-based and machine-learning-driven risk stratification approaches. Future research should focus on correlating these “gold standard” pathological volumes with MRI-derived segmentation parameters, enabling AI tools to non-invasively predict the same high-risk outcomes, such as positive surgical margins or occult lymph node micro-metastases, prior to surgical intervention. From a clinical decision-making perspective, the most actionable application of tumour volumetry would be in the preoperative setting rather than after radical prostatectomy. In the present study, TVP was derived from the surgical specimen and therefore represents a postoperative pathological metric rather than a directly deployable preoperative tool. We used pathological TVP as a reference-standard measure of tumour burden, and this approach may provide a biological benchmark for future studies investigating whether mpMRI-derived tumour volume or imaging-based TVP can reproduce similar associations with adverse pathological features, particularly modal metastasis.

The ROC analysis further supports the biological relevance of tumour burden, as TVP demonstrated moderate discrimination for positive surgical margins and stronger performance for nodal metastasis, indicating that increasing tumour volume percentage may capture aspects of metastatic potential beyond traditional clinical variables. The discrepancy in AUC values between lymph node metastases (0.793) and surgical margins (0.655) offers a crucial biological insight. The higher AUC for nodal disease suggests that tumour volume is a reliable surrogate for metastatic potential. In contrast, the lower AUC for surgical margins implies that “resectability” is not strictly linear. A small apical or anterior lesion in a surgically challenging location may result in a positive margin, whereas a larger mid-gland tumour may still be completely resected. Thus, local tumour topography and resectability may be more relevant drivers of surgical margin status than TVP alone [36,37].

This study has limitations: a single-center design, retrospective analysis, and a lack of long-term biochemical recurrence endpoints in the present report. The biochemical recurrence rate, metastasis-free survival, and overall survival were not reported in the provided data and should be the focus of future analysis. This study is further limited by the low number of extended pelvic lymph node dissections, which restricted the ability to perform template-specific oncologic analyses, and the small absolute number of pN1 cases within this subgroup precluded a reliable statistical comparison between limited and extended PLND templates. In addition, the use of both limited and extended pelvic lymph node dissection templates may have influenced the detection of nodal metastases, introducing potential ascertainment bias in the analysis of pN1 disease. While the cohort size is adequate for multivariate analysis, the findings require validation in larger, multi-institutional cohorts. Still, our internal and external consistency with several cohorts supports the robustness of the conclusion. As trials and registries increasingly incorporate volumetric annotation, harmonized definitions and standardized thresholds (e.g., 2.8 cc for adverse biology in some series [25]) will be crucial to refine and generalize clinical use [38,39].

Furthermore, our data highlight a critical integrity check between preoperative risk assessment and final pathological findings. Although biopsy histology showed that 75.4% of our patients (120/159) presented with ISUP grade groups 1 or 2, the final surgical specimens revealed that 79.3% of the cohort (126/159) had non-organ-confined disease, staged at ≥pT3a. This substantial rate of pathological upstaging suggests that while initial biopsy findings and the median PSA of 13.2 ng/mL might appear to reflect lower-risk disease, these patients correctly met the “high-risk” definition primarily through advanced clinical staging (e.g., ≥cT2c). The discrepancy between biopsy and final pathology reinforces further evaluation of TVP as a quantitative biomarker to more accurately reflect the intrinsic biological aggressiveness and spatial extent of the tumour that categorical biopsy grades may under-represent.

Therefore, the findings of this study should be interpreted as demonstrating cross-sectional associations between tumour volume percentage and adverse pathological characteristics, rather than establishing TVP as a validated prognostic biomarker for long-term oncologic outcomes.

From a clinical perspective, tumour volume percentage represents a quantitative measure of intraprostatic tumour burden that parallels adverse pathological characteristics observed at radical prostatectomy. Although the present study does not evaluate long-term oncologic outcomes, these findings suggest that incorporating tumour volumetric metrics may enhance the characterization of disease aggressiveness when interpreted alongside established clinicopathological parameters.

Importantly, the present findings should be interpreted as demonstrating associations between tumour volume percentage and adverse pathological features at the time of prostatectomy, rather than establishing TVP as an exploratory prognostic marker capable of independently predicting oncologic outcomes.

## 5. Conclusions

Our results suggest that tumour volume percentage was associated with multiple adverse pathological features in high-risk prostate cancer, including advanced pathological stage, higher grade disease, positive surgical margins, and lymph node involvement, rather than perioperative or patient-related characteristics. This reinforces its importance in reflecting intraprostatic tumour burden and may complement established pathological variables when characterizing disease aggressiveness at the time of surgery. TVP may have clinical relevance in integrating TVP into existing prognostic nomograms to improve the accuracy of postoperative risk stratification, especially in high-risk prostate cancer.

## Figures and Tables

**Table 1:** Comparative analysis of clinical and pathological variables and their statistical univariate association with tumour volume percentage.

		TVP (IQR)%	*p*
Age (Years)	66 (IQR: 50–76)		0.041
PSA (ng/mL)	13.2 (IQR: 0.81–41)		0.005
Biopsy ISUP			<0.001
1	23 (14.4%)	3.91 (0.25–14.8)	
2	97 (61%)	7.07 (0.38–29.18)	
3	21 (13.2%)	11.94 (2.36–23.01)	
4	12 (7.5%)	12.02 (1.42–17.27)	
5	6 (3.7%)	15.79 (7.02–32)	
Biopsy Invasion			<0.001
Without	104 (65.5%)	6.81 (0.25–27.43)	
Perineural	48 (30.1%)	8.63 (0.58–31.8)	
Extraprostatic extension	7 (4.4%)	11.89 (3.03–32)	
Total Biopsy Cores	12 (IQR: 6–21)		0.901
Positive Biopsy Cores	6 (IQR: 1–15)		<0.001
Prostatectomy ISUP			<0.001
1	6 (3.77%)	1.18 (0.25–3.07)	
2	94 (59.1%)	7.41 (0.30–29.12)	
3	38 (23.8%)	7.54 (1.15–30.1)	
4	4 (2.5%)	8.94 (3.27–13.76)	
5	17 (10.6%)	9.52 (4.53–32)	
Prostatectomy pT			<0.001
pT2c	60 (37.7%)	6.56 (0.25–29.18)	
pT3a	61 (38.3%)	8.55 (0.4–28.76)	
pT3b	36 (22.6%)	7.98 (0.30–32)	
pT4	2 (1.2%)	15.02 (8.01–22.3)	
Surgical Margin			0.005
Clear	98 (61.7%)	6.06 (0.25–27.32)	
Positive	61 (38.3%)	9.78 (0.58–31)	
Lymph-Node Involvement			0.006
Negative	148 (93.09%)	7.50 (0.25–29.78)	
Positive	11 (6.91%)	12.02 (3.2–32)	
Surgery Duration (minutes)	220 (IQR: 150–360)		0.901
Approach			
Open	82 (51.5%)	7.95 (0.85–29.5)	0.770
Laparoscopic	77 (48.5%)	7.06 (0.25–32)	
Total Hospitalisation (days)	6 (IQR: 4–21)		0.346
ICU Duration (days)	2 (1–6)		0.502

**Table 2:** Multivariable regression analysis after adjustment for clinical and pathological predictors of TVP after radical prostatectomy.

Covariate	β Coefficient	95% CI	*p* Value
Age	−0.128	(−0.328–0.072)	0.208
Hospitalisation days	0.081	(−0.160–0.321)	0.508
Surgery duration	0.005	(−0.012–0.022)	0.608
ICU days	0.16	(−0.711–1.03)	0.363
PSA	0.059	(−0.057–0.175)	0.314
Number of positive lymph nodes	2.59	(1.689–3.508)	<0.001
Positive surgical margin size	1.19	(0.627–1.757)	<0.001
Time from biopsy to surgery	0.002	(−0.009–0.012)	0.310
Prostate Biopsy ISUP			
2	0.75	(−2.07–3.58)	0.600
3	2.81	(−1.09–6.72)	0.157
4	1.61	(−0.97–7.31)	0.254
5	1.81	(−4.38–8.01)	0.563
Invasion			
Perineural	1.77	(−0.41–3.94)	0.111
Extraprostatic extension	8.23	(3.62–12.85)	0.001
Prostatectomy pT stage			
2c	−7.13	(−9.51–−4.75)	<0.001
3a	−5.77	(−8.13–−3.42)	<0.001
3b	−3.37	(−5.86–−0.88)	0.008
4	24.70	(17.69–31.70)	<0.001
Prostatectomy ISUP			
2	3.44	(−1.49–8.37)	0.170
3	2.60	(−2.67–7.87)	0.332
4	−0.73	(−8.63–7.18)	0.332
5	6.60	(0.96–12.25)	0.022

## Data Availability

The original contributions presented in this study are included in the article. Further inquiries can be directed to the corresponding author.
